# Comparison of percutaneous endoscopic thoracic decompression and posterior thoracic laminectomy for treating thoracic ossification of the ligamentum flavum: a retrospective study

**DOI:** 10.1186/s12893-022-01532-z

**Published:** 2022-03-04

**Authors:** Feng-Kai Yang, Peng-Fei Li, Chen-Tao Dou, Rong-Bo Yu, Bin Chen

**Affiliations:** Department of Minimally Invasive Spine Surgery, Affiliated Hospital of Chengde Medical University, Chengde, 067000 Hebei China

**Keywords:** Ossification of the ligamentum flavum, Thoracic myelopathy, Thoracic spinal stenosis, Percutaneous endoscopic thoracic decompression, Posterior thoracic laminectomy

## Abstract

**Background:**

Thoracic spinal stenosis (TSS) caused by ossification of the ligamentum flavum (OLF) is generally treated by surgical decompression. In this study, we compared the efficacy and safety of percutaneous endoscopic thoracic decompression (PETD) and posterior thoracic laminectomy (PTL) for treating thoracic ossification of the ligamentum flavum (TOLF).

**Methods:**

Twenty consecutive patients with TSS caused by TOLF who were treated between April 2016 and May 2020 were included in this retrospective study. They were divided into the PETD (n = 11) and PTL (n = 9) groups. The mean follow-up period was 19.6 months. The visual analogue scale (VAS) score, the modified Japanese Orthopedic Association (mJOA) score and the recovery rate (RR) were used to evaluate the clinical outcomes.

**Results:**

There were significant differences between PETD group and PTL group in operative time (min) (95.0 ± 18.8 vs 131.1 ± 19.0), postoperative drainage (mL) (20.2 ± 7.9 vs 586.1 ± 284.2), hospital stay (days) (4.4 ± 1.2 vs 10.4 ± 2.6) (P < 0.05 for all). However, both groups had similar and significant improvement in VAS and mJOA scores. The RR of two groups achieved the same improvement (81.8% VS 77.8%, P > 0.05).

**Conclusions:**

The use of PETD and PTL for treating TOLF both achieved favorable outcomes. PETD is both minimally invasive and achieves similar postoperative symptom relief to PTL. Therefore, PETD could be considered as an effective alternative to traditional open surgery for TOLF in single-segment lower thoracic spine.

## Background

Thoracic myelopathy is mainly caused by ossification of the ligamentum flavum (OLF) compressing the spinal cord. This causes lower limb numbness and weakness and bladder and bowel dysfunction, which seriously impacts patients’ quality of life [1]. Thoracic ossification of the ligamentum flavum (TOLF) is particularly common in East Asian countries such as China, Japan, and Korea, and it is the leading cause of thoracic spinal stenosis (TSS) [2, 3]

Once neurological symptoms occur in patients, conservative treatment is generally ineffective, and spinal stenosis and cord compression can be progressive. The longer the time from symptom onset to surgical treatment, the worse the prognosis [4]. Therefore, surgical treatment should be initiated as early as possible once imaging findings confirm the cause of the clinical symptoms. At present, the most common surgical treatment in clinical settings is posterior thoracic laminectomy (PTL), involving extensive dissection of paraspinal muscles and removal of the spinous process, lamina, and hyperplastic ligament [5]. However, there can be severe intraoperative complications such as deterioration of spinal cord function.

How to treat TOLF with a less invasive, simple, safe, and effective method to relieve the patients’ suffering and improve their quality of life is an urgent clinical problem [6]. In recent years, percutaneous endoscopic spinal surgery has achieved remarkable results for treating disc herniation [7]. As the endoscopic technique has matured, its indications have been broadened to the treatment of spinal stenosis [8]. Due to the limited reserve space of the thoracic spinal canal, the thoracic spinal cord is very sensitive, and the operating space is limited by the ribs. Few reports detail the success of percutaneous endoscopic thoracic decompression (PETD) for treating TOLF [9, 10]. The purpose of this study was to compare the efficacy and safety of PETD versus PTL for treating TOLF.

## Materials and methods

### Patient population

From April 2016 to May 2020, 20 consecutive patients underwent surgery for single-segment TOLF, the patients were divided into PETD group (n = 11) and PTL group (n = 9) according to different surgical methods. There was no significant difference in sex, age, location and classification of the ossification between the PETD and PTL groups. The inclusion criteria were as follows: (1) diagnosis of spinal stenosis due to TOLF confirmed by MRI and CT and (2) typical symptoms of thoracic myelopathy. The exclusion criteria were as follows: (1) thoracic lumbar disc herniation or other severe spinal disease; (2) previous spinal trauma or surgery, severe cardiopulmonary disease; and (3) incomplete case information or loss to follow-up.

This retrospective study was approved by the Ethics Committee of Chengde Medical University Affiliated Hospital (CYFYLL2021094). Informed consent was obtained from all patients. The percutaneous transforaminal endoscopic spine system (outer diameters of endoscope: 7.3 mm, Joimax GmbH, Karlsruhe, Germany) and bipolar radiofrequency system (Elliquence LLC, Baldwin, NY, USA) were used in PETD.

### Surgical procedure

#### PETD

**Fig. 1:**
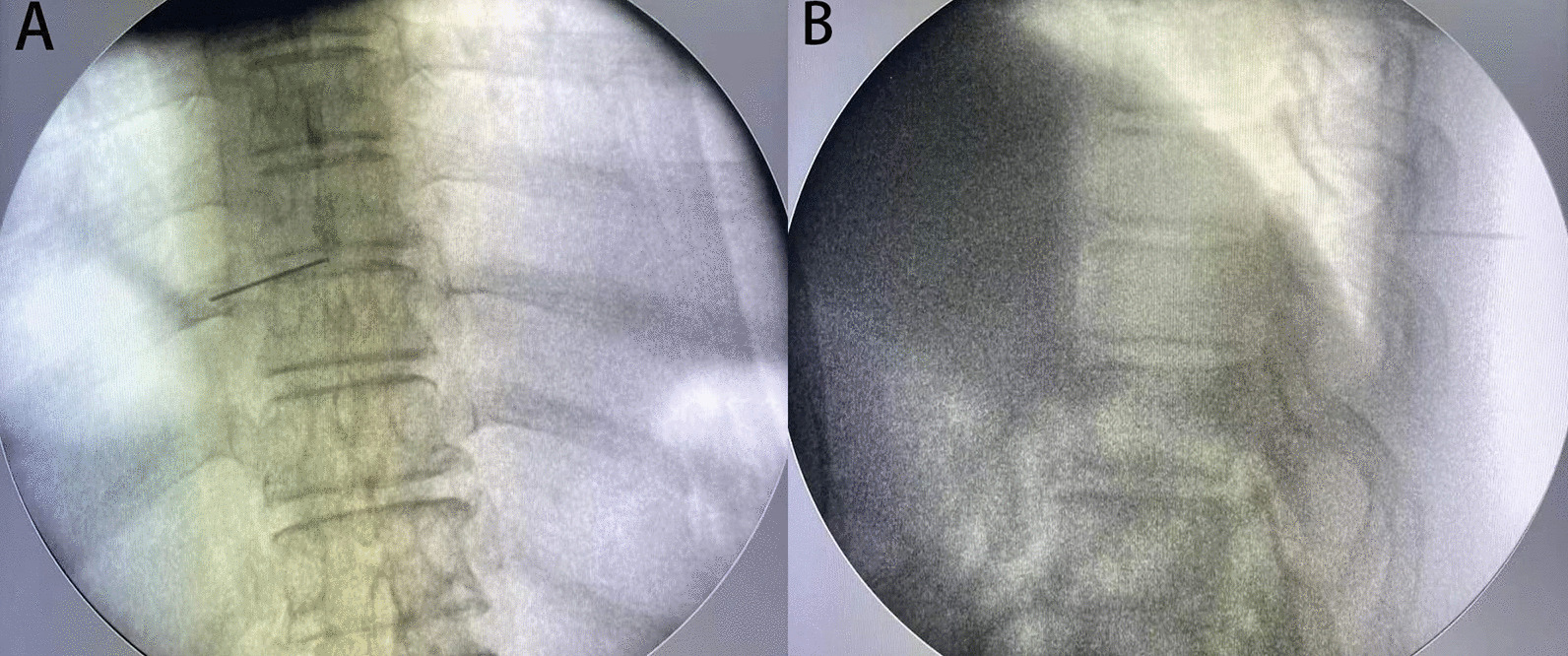
The spinal needle was placed on the medial margin of the facet joints in fluoroscopic views. **A** In anteroposterior view. **B** In lateral view

**Fig. 2:**
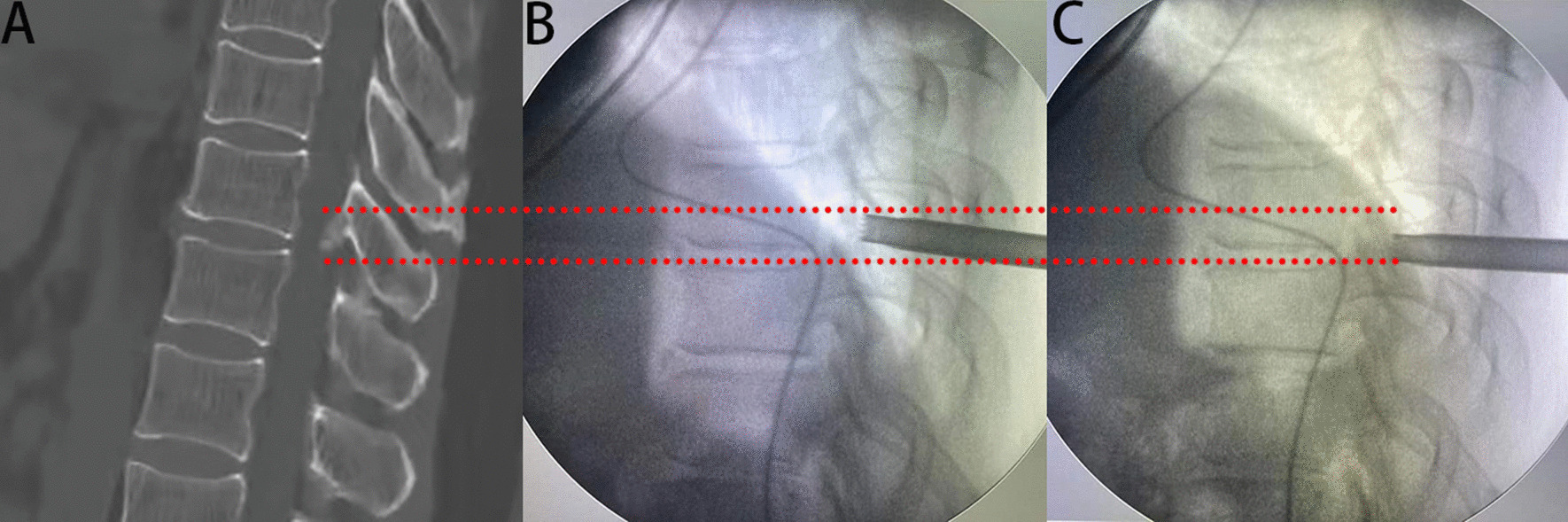
**A** Preoperative CT (sagittal). **B** Mark the cranial boundary of ossification on the surface of the lamina with the trephine. **C** Mark the caudal boundary of ossification on the surface of the lamina with the trephine. Dotted line; Roughly delimit the removal range of the lamina

**Fig. 3:**
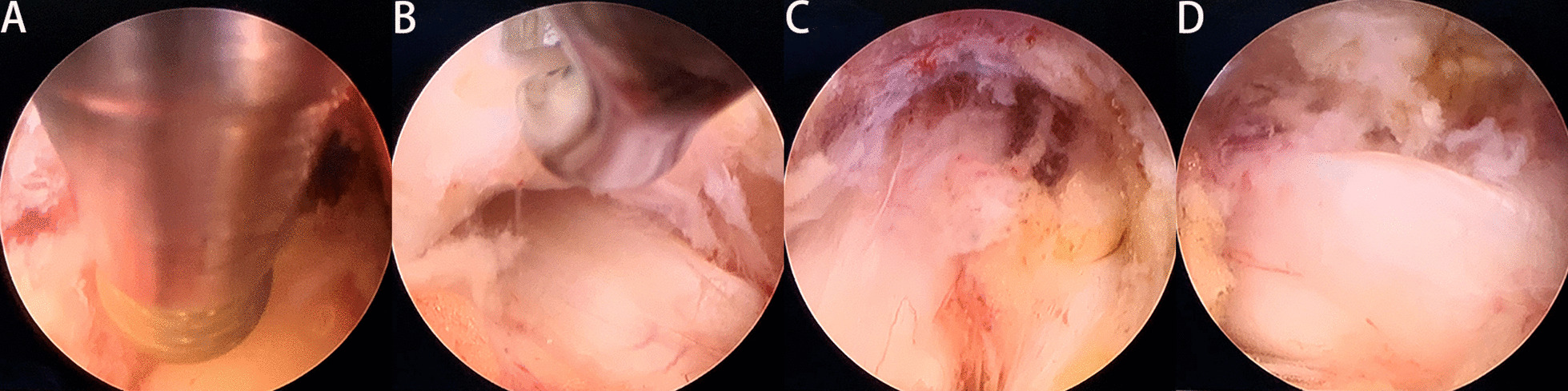
Intraoperative endoscopic views. **A** The high-speed drill grinding the lamina. **B** The thinned OLF removed by nucleus pulposus forceps. **C** The border of the spinal cord was clearly visible, and the compression had been relieved. **D** The contralateral spinal cord boundary was also visible and the spinal dura sac fluctuated well

Surgery was performed under local anesthesia and the more severe side was selected as the approach side. Patients were placed in a prone position on a radiolucent table. The puncture entry point was 2–4 cm lateral to posterior midline. Under fluoroscopic guidance, a spinal needle was introduced after infiltration of local anesthetic (1% lidocaine). After the needle came into contact with the lamina, the tip was positioned at the medial margin of the facet joints on the anteroposterior fluoroscopic projection (Fig. 1). A guidewire was introduced into the needle. A skin incision of approximately 7–8 mm was then made and dilators were sequentially passed along the guidewire. Thereafter, a bevelled working cannula and a trephine were inserted. The final position was based on preoperative imaging and intraoperative fluoroscopy. Marks were made on the surface of the lamina using the trephine under fluoroscopic guidance, in order to roughly delimit the removal range regarding the caudal and cranial sides (Fig. 2). After removing the tissue on the surface of the lamina under endoscopic visualization, the lamina was completely exposed. A high-speed drill was then used to grind away the lamina to expose the ipsilateral OLF, using the marks made by the trephine as a guide (Fig. 3A). After the ossified ligament was thinned (so that it was nearly translucent) using a diamond drill, it was carefully removed using nucleus pulposus forceps (Fig. 3B). The angle of abduction was appropriately increased to perform contralateral decompression in the same manner. Finally, the exposed dural sac was checked to confirm that the pulsation was improved (Fig. 3C, D). After decompression, all endoscopic instruments were removed, and the incision was closed with a suction drainage.

#### PTL

PTL was performed according to a previous study [11] under general anesthesia. Routine treatments were same as PETD group, such as anti-inflammatory drugs and neurotrophic agents were given after surgery to decrease edema and inflammation.

### Measures

Pre- and postoperative Neurological status were assessed using the visual analogue scale (VAS) and the modified Japanese Orthopaedic Association (mJOA) score. Recovery rate (RR) was calculated using the following formula: RR = 100% × (postoperative − preoperative mJOA score)/(11 − preoperative mJOA score). According to RR, clinical outcomes were divided into excellent (75–100%), good (50–74%), fair (25–49%) and poor (0–24%) [12].

### Statistical analyses

The SPSS Version 25 software (IBM, Armonk, New York, USA) was used for all statistical analyses. Patients’ demographic data and perioperative outcomes were performed using unpaired t-test and fisher's exact test. For a comparison of mJOA between the two groups, repeated-measures analysis of variance was used to evaluate the data. P < 0.05 was considered statistically significant.

## Results

The demographic characteristics of patients are presented in Table 1. Perioperative parameters of two groups including intraoperative blood loss, operative time, postoperative drainage and hospital stay were summarized in Table 2. Compared with the PTL group, the PETD group had advantages in operative time (minutes) (95.0 ± 18.8 vs 131.1 ± 19.0, P < 0.05), postoperative drainage (mL) (20.2 ± 7.9 vs 586.1 ± 284.2, P < 0.05), hospital stay (days) (4.4 ± 1.2 vs 10.4 ± 2.6, P < 0.05).

**Table 1:** Preoperative demographic data

Characteristics	PETD group (n = 11)	PTL group (n = 9)	P value
Age (years)	61.9 ± 7.1	60.8 ± 7.2	0.73
Sex (male)	8	3	0.18
Duration of symptoms (months)	10.5 ± 8.4	8.1 ± 9.2	0.55
Body mass index (kg/m^2^)	23.8 ± 1.5	23.4 ± 1.3	0.62
Level			0.11
T8–9	0	1	
T9–10	2	0	
T10–11	7	3	
T11–12	2	5	
Sato classification			0.93
Lateral type	3	3	
Extended type	3	2	
Enlarged type	4	2	
Fused type	1	2	
Tuberous type	0	0	
Dural adhesion	3	2	
Dural ossification	1	0	

*PETD* percutaneous endoscopic thoracic decompression; *PTL* posterior thoracic laminectomy

**Table 2:** Perioperative parameters

Characteristics	PETD group (n = 11)	PTL group (n = 9)	P value
Duration of surgery (minutes)	95.0 ± 18.8	131.1 ± 19.0	0.00
Drainage (mL)	20.2 ± 7.9	586.1 ± 284.2	0.00
Hospital stay (days)	4.4 ± 1.2	10.4 ± 2.6	0.00
Complications	3/11	2/9	

*PETD* percutaneous endoscopic thoracic decompression; *PTL* posterior thoracic laminectomy

Follow-up for neurological status after surgery assessed by VAS, mJOA and RR. The evaluation of preoperative neurological status showed that VAS and mJOA scores were similar between PETD group and PTL group (5.9 ± 1.0 vs 6.0 ± 0.7, P = 0.74; 6.1 ± 1.2 vs 5.9 ± 1.4, P = 0.73). The mean duration of clinical follow-up was 18.4 ± 9.4 months in the PETD group and 21.0 ± 8.1 months in the PTL group. All patients had been followed up for more than 6 months. The mean VAS and mJOA scores of both groups had similar improvement during the postoperative follow-up. The postoperative VAS scores in the PETD group and PTL group improved at 1 months (2.6 ± 0.9 and 2.8 ± 1.0, P = 0.00), and final follow-up (2.0 ± 0.6 and 2.1 ± 0.8, P = 0.00). The postoperative mJOA scores in the PETD group and PTL group also improved significantly at 1 months (8.0 ± 1.0 and 7.8 ± 1.1, P = 0.00), 3 months (8.5 ± 0.8 and 8.2 ± 1.1, P = 0.00), 6 months (9.2 ± 1.1 and 8.8 ± 1.1, P = 0.00), and final follow-up (9.5 ± 1.1 and 9.2 ± 0.8, P = 0.00). In addition, there were no significant differences in VAS and mJOA scores at each follow-up time point between groups (P > 0.05). At the final follow-up, the good-to-excellent rate in PETD group and PTL group were 81.8% and 77.8%. There were no statistically significant differences between groups (P = 0.74).

### Complications

Cerebrospinal fluid leakage and neurological deterioration are common postoperative complications. The rate of cerebrospinal fluid leakage in related studies was 13–29%, and it was more common in patients with dural adhesion or dural ossification [13]. In this study, one patient in the PETD group had an intraoperative dural tear, but without cerebrospinal fluid leakage. We directly covered the tear with gelatin sponge and there were no other complications after the operation. Two patients developed transient paralysis immediately with lower limbs limited movement and sensory disturbances after decompression and both recovered within 24 h. In the PTL group, two patients developed postoperative cerebrospinal fluid leakage, one of whom also had incision infection. Although the recovery was good after symptomatic treatment, it necessitated prolonged postoperative rehabilitation.

## Discussion

The effective volume of the thoracic spinal canal is relatively narrow compared to that of the lumbar spinal canal. If there is anterior or posterior compression, there is almost no buffer space, and the compensatory ability is poor. Additionally, the thoracic spinal cord has a significantly lower blood supply than the cervical and lumbar segments, and less collateral circulation. Postoperative neurological deterioration is related to intraoperative injury of blood vessels around the spinal cord [13]. Owing to these anatomical and blood supply characteristics, the thoracic spine was once considered a restricted area regarding spinal surgery. Reported rates of postoperative neurological deficit range from 5.7 to 33% about thoracic spine surgery [14–16].

TOLF mostly occurs in the lower thoracic spine. Low back pain and numbness of the lower limbs can be the main clinical manifestations in early-stage TOLF, which can be mistakenly attributed to lumbar spine disease, thus reducing the chance of early diagnosis. When there is progressive neurological dysfunction, early decompression should be performed to prevent irreversible spinal cord damage. The location of the compression, number of segments involved, and general patient condition should be used to determine an appropriate treatment plan.

PTL is usually used to relieve posterior compression, which is mainly caused by OLF [17, 18]. Although laminectomy achieves good spinal cord decompression, excessive removal of the lamina and facet joint can cause thoracic instability [19]. Therefore, pedicle screw fixation and fusion are sometimes required. Kim et al. [20] reported successful bilateral decompression using unilateral laminectomy to treat 11 cases of TOLF. The recovery rate was 33.2%. Although no fusion was performed, the rates of postoperative thoracic kyphosis and instability did not increase. However, laminectomy inevitably comes at the price of complications such as increased rates of acute neurological deterioration and dura tears. Improper use of a laminar rongeur or an osteotome can also cause spinal cord concussion or other spinal cord injury.

Some researchers have recommended treating TOLF with laminoplasty, which achieves nerve decompression by expanding the volume of the spinal canal without removing the ligamentum flavum. Because most of the posterior structure of the spinal canal is retained, there is little effect on spinal stability. However, it is not recommended for severe OLF because the expandable space is limited and lamina reclosure can occur [21]. The current most widely used surgical technique for treating TOLF remains laminectomy with or without fusion [22].

Given the sensitivity of the thoracic spinal cord and the special anatomy of the thoracic spine, it is necessary to develop an effective surgical technique that is less traumatic, which could ensure fewer complications. In recent years, endoscopic techniques have been used for cervical and lumbar spine surgery, and they have achieved good clinical results. The concept of minimally invasive surgery is not new, but it needs to be emphasized that the surgery involves reduced invasiveness while still ensuring efficacy. Both Jia et al. [8] and Miao et al. [9] reported successful treatment of TOLF using PETD. An et al. [23] performed PETD to treat 18 patients with various types of TOLF. At a mean follow-up point of 17.4 months, the recovery rate was 47.5% and the modified Japanese Orthopedic Association (mJOA) score was obviously improved.

**Fig. 4:**
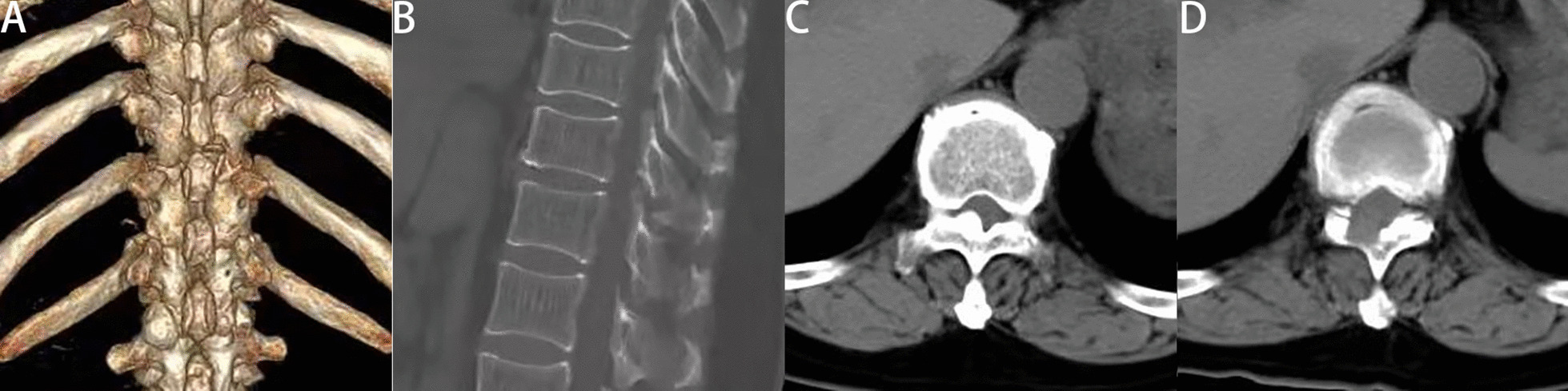
**A** Postoperative 3D reconstruction showed the location of the working channel. Compared with the preoperative CT (Fig. 2A), the postoperative CT (sagittal) (**B**) revealed that the OLF was completely removed. Computed tomography of the thoracic spinal cord showed that adequate decompression was performed. **A** Preoperative CT (axial). **B** Postoperative CT (axial)

**Fig. 5:**
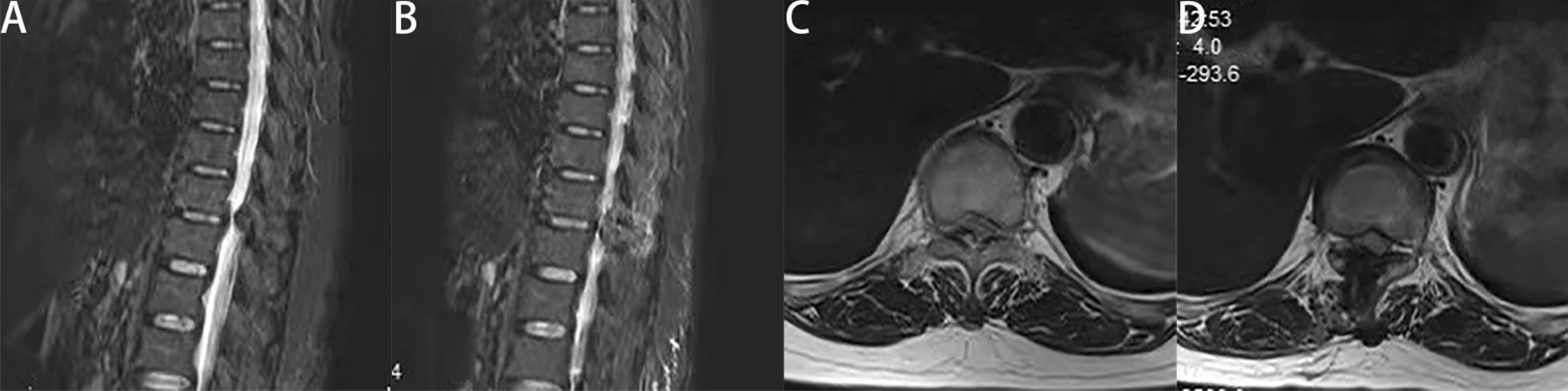
Satisfactory decompression was assessed by comparing the preoperative (**A**, **C**) and postoperative (**B**, **D**) MRI

Endoscopic spinal surgery has unique advantages for the treatment of TOLF. It can be performed under local anesthesia. Most traditional surgery uses general anesthesia, and some intraoperative neuromonitoring is performed. However, studies have shown that intraoperative neuromonitoring does not reduce neurological complications [24]. Under local anesthesia, the patient is conscious and able to communicate with the surgeon at any time. When the spinal cord or nerve roots are touched during the operation, the patient may have pain or numbness, and the surgeon can immediately stop the operation to avoid nerve damage. Especially for elderly patients for whom general anesthesia is unsuitable, PETD may be a solution worth considering. In our study, the spinal canal is fully decompressed and the preoperative symptoms are relieved (Figs. 4, 5). No patient discontinued the surgery due to pain or psychological stress. In most cases, intervertebral fusion is not necessary and medical expenses are therefore reduced. To evaluate the approximate decompression range, and especially to determine the removal range regarding the caudal and cranial sides of the lamina, we used a trephine to clearly mark the surface of the lamina under fluoroscopic guidance. This made judging the decompression boundary under endoscopic visualization easier. In addition, the spinal endoscopy technology magnified the field of vision. During the operation, radiofrequency coagulation of small blood vessels and bleeding points was used to ensure a clear field of vision. This helped to accurately remove the lesion and reduce damage to surrounding soft tissues. Using a diamond high-speed drill with continuous saline irrigation reduced local high temperature. After thinning the OLF, it was removed using nucleus pulposus forceps. However, the spinal cord might still be accidentally irritated during this process. In this study, two patients developed transient paralysis in surgery, and recovered after glucocorticoid therapy. Dural adhesion and dural ossification increase the risk of dural tear and the difficulty of surgery [25, 26]. In the PETD group, dural adhesion was found in 3 patients during operation, and one of them had dural tear. One patient was accompanied by dural ossification. After thinning and floating the ossified dural, we did not forcibly remove it, which did not affect the postoperative recovery. Two patients in the PTL group experienced dural tear with cerebrospinal fluid leakage due to dural adhesion.

The use of PETD to treat TOLF requires the surgeon to be skilled in spinal endoscopic decompression, and the learning curve is relatively steep. Regrettably, we were not able to provide quantitative measurements for facet removal between the two groups due to radiographic data was not complete enough. The limitations of this retrospective study are the small sample size and lack of longer follow-up. To objectively and comprehensively evaluate the safety and effectiveness of this method, more patients need to be recruited for multicenter, randomized controlled trials in the future.

## Conclusion

The use of PETD and PTL for treating single-segment TSS caused by OLF in lower thoracic spine both achieved satisfactory outcomes. Although there are few relevant clinical reports at present, we believe that PETD is an effective surgical method for treating TOLF. PETD enriches the surgical treatment methods for TOLF and helps to formulate an appropriate treatment plan for each individual patient.

## Data Availability

The datasets used and/or analysed during the current study are available from the corresponding author on reasonable request.

## Abbreviations

TSS: Thoracic spinal stenosis
OLF: Ossification of the ligamentum flavum
PETD: Percutaneous endoscopic thoracic decompression
PTL: Posterior thoracic laminectomy
TOLF: Thoracic ossification of the ligamentum flavum
VAS: The visual analogue scale
mJOA: Modified Japanese Orthopedic Association
RR: Recovery rate
MRI: Magnetic resonance imaging
CT: Computed tomography
